# Systematic Review of the Relationship Between Autism Stigma and Informal Caregiver Mental Health

**DOI:** 10.1007/s10803-018-3835-z

**Published:** 2018-12-19

**Authors:** Chris Papadopoulos, Annemarie Lodder, Georgina Constantinou, Gurch Randhawa

**Affiliations:** University of Bedfordshire, Putteridge Bury Campus, Hitchin Road, Luton, LU2 8LE UK

**Keywords:** Autism, Stigma, Mental health, Caregiver, Carer, Theoretical framework

## Abstract

Families play a crucial role in determining the mental health of the autistic individual(s) they are caring for. However, the stigma associated with autism can impair caregiver health. To investigate this, empirical evidence pertaining to stigma’s impact on informal caregivers’ mental health was systematically reviewed. All twelve included studies (n = 1442 informal caregivers) consistently reported the impact of autism related stigma upon caregiver mental health to be significant, meaningful and complex. A new theoretical framework describing the relationship between stigma and caregiver mental health is constructed. Moderating variables include those both changeable through intervention (e.g. hopelessness, self-esteem, self-compassion) and not changeable (gender, culture, financial burden and time since diagnosis). Implications and recommendations for professionals, interventions and future research are proposed.

## Introduction

Informal caregivers of autistic individuals (i.e. people who provide unpaid care and support to a family member in a non-professional capacity) are at a substantially elevated risk of poorer mental health compared to the general population (Da Paz and Wallander 2017; Mugno et al. 2007; Isa et al. 2013; Samuel et al. 2012; Zablotsky et al. 2013; Sander and Morgan 1997). When informal caregivers experience poor mental health, the individuals they care for are at an increased risk of making delayed developmental progress (Osborne et al. 2008). This may not be surprising given that caregivers play a crucial role in influencing their mental health (Dykens et al. 2014).

One of the key contributory factors for poor mental health among informal caregivers is the stigma associated with autism and the complex influence this has. This has been highlighted by Kinnear et al. (2015) whose study of 502 families of autistic children determined that the difficulties associated with autism stigma play a crucial role in how difficult life will be for parents overall. The consequences of public stigma also include autistic children being more vulnerable to experiencing social rejection and loneliness compared to typically developing children (Kinnear et al. 2015; Bauminger and Kasari 2000; Bauminger et al. 2003).

Autism stigma also extends to informal caregivers in the form of ‘courtesy stigma’, that is, the stigma experienced by individuals who are closely associated with the individuals with a stigmatic mark. This type of stigma includes the blaming of caregivers for the onset of autism and deterioration of their child’s development, an expectation that they should be ashamed, that they lack competence in their caregiving role, and that it is best if they are avoided and/or pitied (Milacic-Vildojevic et al. 2012). Holding such views can lead to caregivers being socially rejected (Gray 1993; Corrigan et al. 2006).

Informal caregivers are also vulnerable to ‘affiliate stigma’ (Mak and Cheung 2008). This is when the public’s negative stereotypes towards both autistic individuals and their caregivers subsequently become accepted by caregivers and incorporated within their own psychological identity (Papadopoulos 2016a). Informal caregivers then ‘self-stigmatise’ themselves, consequently becoming more vulnerable to feeling unhappy (Mak and Cheung 2008), being less likely to cope with adversity (Jahoda and Markova 2004; Meyers et al. 2009) and being more likely to perceive themselves and their situation negatively (Corrigan and Watson 2002). According to Mak and Cheung (2008), informal caregivers who experience self-stigma react behaviourally by concealing their status from others, withdrawing from social relations, and may even alienate themselves from the targeted individuals to avoid association. This links with the ‘why try’ effect; a psychological response to self-stigma which reduces one’s motivation towards achieving life goals and accessing support (Corrigan et al. 2009). This increases the risk of concealing their situation from others and withdrawing from social events (Mak and Cheung 2008). Therefore, affiliate stigma produces negative cognitive, affective and behavioural responses that serve to damage mental health, quality of life and place extra strain on the caregiving relationship. If caregivers experience mental health problems, then they may experience a double-stigma since mental health problems remain highly stigmatised (Papadopoulos 2016b).

This systematic review aims to identify and synthesise existing empirical evidence associated with autism-related stigma in its various forms and the influence this has upon the mental health of informal caregivers of autistic individuals. This can help to establish clarity regarding the strength and nature of these relationships, and to identify and prioritise areas for further research including areas of focus for future interventions. A secondary aim was to construct a new theoretical framework based upon the evidence found pertaining to the relationship between autism stigma and informal caregiver mental health.

## Methods

### Inclusion Criteria

The study inclusion criteria were as follows: primary studies or secondary statistical analysis studies that reported any empirical data associated with the relationship between autism-related stigma and mental health among informal caregivers of autistic individuals. Therefore, studies that had sampled caregivers of individuals with different types of disabilities were only included if they reported upon stigma and its relationship with mental health among caregivers of autistic people specifically. Peer-review journal articles; studies published in the English or Dutch language; and studies published between January 1990 and June 2018 were the other inclusion criteria. Studies that did not meet all criteria were excluded. Similar to the conceptual approach of Zuckerman et al. (2018) and Yu et al. (2016), we included studies that reported any subjective and objective data on established mental health concepts including general mental health, psychological and emotional distress and wellbeing, depression, anxiety, obsessive compulsive disorder, post-traumatic stress disorder, suicidal ideation, and psychotic disorders. However, broader, related concepts (that are less likely to be considered mental health conditions per se) including general stress, parental strain, difficulty of raising an autistic child, loneliness, and feelings of hope, shame and embarrassment were excluded as outcomes of interest.

### Search Strategy

After obtaining ethics approval from the Institute for Health Research Committee, the following searching keyword strategy was applied on the electronic database, PubMed, PsycInfo, CINAHL, ScienceDirect, ERIC and SOCIndex: (stigma* OR attitud* OR prejudic* OR discriminat* OR judgement* OR stereotyp* OR belief* OR views*) AND (carer* OR caregiv* OR famil* OR parent* OR sibling*) AND (autis* OR asd OR asc OR asperger* OR PDD OR “intellectual disabilit*” OR “learning disabilit*” OR “neurological disabilit*” OR “development* disabilit*”) AND (mental* OR psych* OR well*).

After removing duplicates, all retrieved titles and abstracts were screened by CP and AL. Full-texts of papers that passed the title and abstract stage were sourced and screened independently by CP, AL, and GC. The reference lists of these papers were checked for any potentially relevant literature not previously identified. There were six screening disagreements all of which were resolved by discussion and reaching a shared consensus. Most disagreements centred on whether there was a clear association between autism stigma and informal caregiver mental health, and whether the mental health outcome of interest aligned with the agreed conceptualisation of mental health (described above). The authors of papers were contacted when further information and/or clarity was required for an accurate assessment of eligibility, both in terms of the study itself and particular extracts of reported data (for example, if a quote describing a stigma experience was produced from a caregiver of an autistic individual specifically). The searching process took place between April and June 2018.

### Data Extraction

Using Microsoft Excel, a spreadsheet with the following extraction columns was used: author(s), study title, publication year, location, study aims, study design, sample characteristics, sample size, sampling strategy, response rates, retention rate, type of stigma as defined by authors, type of stigma identified by research team, stigma measurement/assessment information, mental health problem/outcome(s) assessed in relation to stigma, mental health assessment methods, quantitative tools’ validity and reliability, study quality, analytical methods, stigma results, mental health results, stigma and mental health relationship results, and study limitations. AL and GC undertook the data extraction process. CP cross-checked all extracted data to ensure accuracy. Identified discrepancies were discussed and agreements reached.

### Quality Appraisal

The Joanna Briggs Institute Critical Appraisal tools were used to assess study rigour, specifically the ‘Checklist for Qualitative Research’ and ‘Checklist for Prevalence Studies’ (and their associated manuals which includes clear explanations of each appraisal criterion) for the included qualitative and quantitative studies respectively. This exercise was conducted by CP and AL who independently assessed the included studies. Higher summed scores equated to higher study quality. A Cohen’s kappa interrater reliability test showed good rater agreement (κ = .745, p < .001), as did a Spearman’s rho correlation (ρ = .745; p < .001). Disagreements were resolved via team discussion and reaching a shared consensus.

### Analysis

The heterogeneity of study designs, particularly in relation to setting and outcome measurement tools, meant that a meta-analysis of quantitative data was viewed as inappropriate. Further none of the included qualitative studies reported the statistics required for such an analysis. Therefore, narrative synthesis was chosen to best investigate the data, appropriate when synthesizing evidence from different study designs (Lucas et al. 2007). After testing various synthesis options, including synthesis by stigma concepts, mental health issues, and study designs, it was concluded that grouping evidence by socio-cultural setting resulted in the most useful and appropriate basis of comparison and interpretation given the diverse range of settings presented across the included studies and that stigma is itself a socio-cultural phenomenon constructed from ideas of what should and should not be valued.

## Results

### Study Identification

**Fig. 1:**
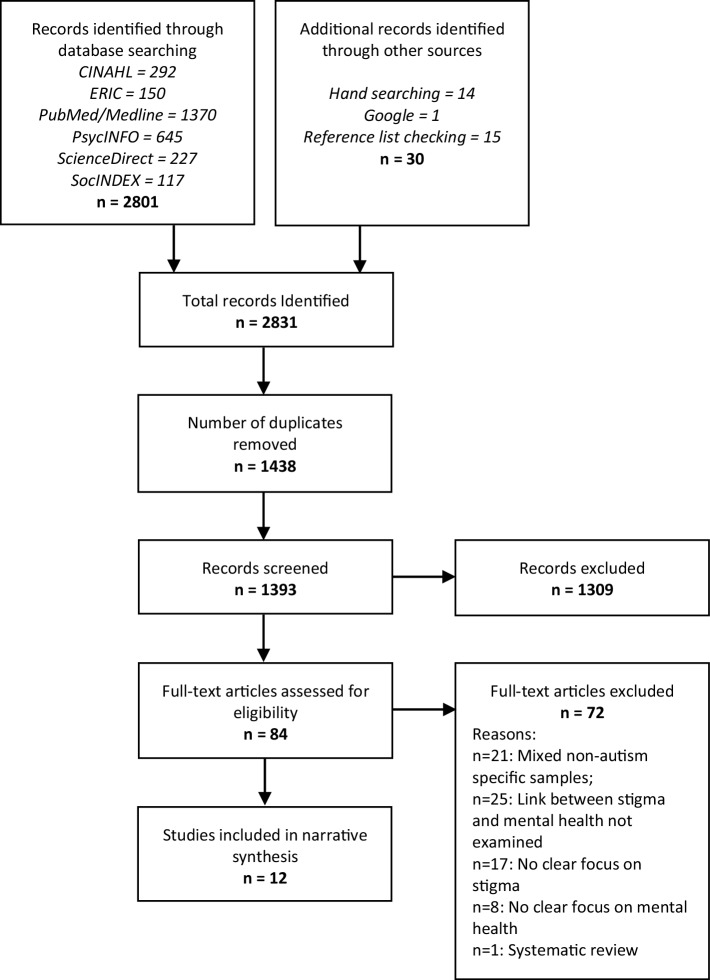
PRISMA flow diagram of study literature identification

The searches retrieved 2,831 articles of which 1438 were duplicates and removed. This left 1393 unique articles of which 84 were retained during the titles and abstracts screening stage. The reasons for exclusion included data reported upon non-autism specific or mixed samples (e.g. Ngo et al. (2012) study in which results pertained to a children with intellectual disabilities with no demarcation of autistic children specifically), no clear empirical relationship between autism-related stigma and caregiver mental health present (e.g. Gray’s (1993) study which describes detailed accounts of stigma but does not examine stigma in relation to mental health), no clear focus, assessment or exploration of autism stigma (e.g. Gatzoyia et al. (2014) study of illness perceptions and their association with parents’ psychological wellbeing) and no clear focus, assessment or exploration of mental health (e.g. Kinnear et al. (2015) who report upon stigma’s association with the ‘overall difficulty’ of raising an autistic child but not stigma’s relationship with mental health per se). Ali et al. (2012) systematic review of self-stigma in people with intellectual disability and courtesy stigma in the family members of people with intellectual disability was also excluded. After completing the full-text screening stage, including manually checking through the references of these articles, 12 articles were identified to meet all criteria and were included into the analysis. A detailed breakdown of the study identification process is presented in Fig. 1.

### Study Characteristics

The included studies represented a total sample of 1442 informal caregivers (predominantly mothers) across three cultural regions: East Asia including China (Ting et al. 2018) and Hong Kong (Chan and Lam 2017, 2018; Mak and Kwok 2010; Wong et al. 2016), the Middle East including Israel (Werner and Shulman 2013), West Bank (Dababnah and Parish 2013), the United Arab Emirates (Crabtree 2007) and Iran (Dehvani et al. 2011), and the Western culture including the United States (Resch et al. 2010) and Australia (Broady et al. 2018 and; Gray 2002).

Studies employed either a qualitative (n = 5) or quantitative (n = 7) methodological approach with none utilising mixed methods. Both Crabtree (2007) and Gray (2002) conducted a longitudinal design with qualitative semi-structured interviews taking place twice with the same participants over a 10 month and 10 year period respectively. Resch et al. (2010) and Dababnah and Parish (2013) both conducted focus groups although the latter also conducted one-to-one semi structured interviews, as did Broady et al. (2018). All seven of the quantitative studies employed a cross-sectional questionnaire survey approach using non-randomised sampling techniques. A full breakdown of the studies’ background information and methodological details, organised by cultural region, is presented in Table 1.

**Table 1:** Background, methodological characteristics and summary of key results of included studies organised by cultural region

Cultural region	Study authors and setting	Key study aim(s)	Study approach and/or design	Sampling method	Sample characteristics	Type(s) of stigma identified by study authors or current authors	Stigma assessment method	Mental health assessment method	Summary of key results
East Asian	Ting et al. (2018); Mainland China	To examine the effects of self-esteem, proneness to shame and family functioning on affiliate stigma and depression with a sample of caregivers of children with autism in Mainland China	Quantitative cross-sectional questionnaire survey	Convenience sampling	263 caregivers, 80.6% of whom were parents, and 15.6% were grandparents. The mean age of participants was 34.6 years (SD = 5.4), 71.5% of caregivers were female, while 74.9% of their children were male. The mean age of participants’ autistic children was 5.39 years (SD = 2.38)	Affiliate stigma	‘Affiliate Stigma Scale’ (Mak and Cheung 2008)	Depression measured by the CES-D (the Center for Epidemiologic Studies Depression scale, Radloff 1977)	Affiliate stigma significantly positively correlated with depression (r = 0.55, p = < 0.01). After integrating results of two path analyses, caregivers low in self-esteem, high in proneness to shame, with poor family adaptability were found to be significantly more likely to experience affiliate stigma and have more depressive symptoms
	Chan and Lam (2017); Hong Kong, China	To test the associations of public stigma and courtesy stigma with depression, anxiety, and caregiving burden among parents of children with ASD and to explore whether trait mindfulness would moderate these associations	Quantitative cross-sectional questionnaire survey	Purposive sampling	424 parents of children with autism, most of whom were mothers (86.1%), married (87.5%), unemployed (63%) and had received a high school level of education (94.1%). Mean age of parents = 43.6 years; mean age of children = 10.41. Majority of children were male (83.3%), while 60.4% had an intellectual disability in addition to autism	Public stigma, courtesy stigma	Public stigma: The ‘Perceived Public Stigma Scale’ which contained 8 items adapted from Green (2001) Courtesy stigma: The ‘Perceived Courtesy Stigma Scale’ which contained seven items adapted from the Devaluation of Consumer Families Scale (Struening et al. 2001)	Depressive symptoms were measured using the 9-item ‘Patient Health Questionnaire’ (Kroenke et al. 2001) Anxiety symptoms were measured using the 7 item ‘Generalized Anxiety Disorder Scale’ (Spitzer et al. 2006)	Public stigma significantly positively correlated with depression (r = 0.27, p = < 0.001) and anxiety (r = 0.23, p = < 0.001) Courtesy stigma significantly positively correlated with depression (r = 0.32, p = < 0.001) and anxiety (r = 0.47, p = < 0.001)
	Chan and Lam (2018); Hong Kong, China	To examine the psychometric properties of a recently developed measure of self-stigma process, the Self-Stigmatizing Thinking’s Automaticity and Repetition Scale (STARS), among parents of children with autism	Quantitative cross-sectional questionnaire survey	Purposive sampling	Same as Chan and Lam (2017)	Self-stigma content and process	Self-stigma content: ‘Affiliate Stigma Scale’ (Mak and Cheung 2008) Self-stigma process: 8 item Self-Stigmatizing Thinking’s Automaticity and Repetition Scale (STARS) (Chan and Mak 2017)	Depressive symptoms were measured using the 9-item ‘Patient Health Questionnaire’ (Kroenke et al. 2001)	Self-stigma content and process both significantly positively correlated with depression (r = 0.45, p = < 0.001 and r = 0.61, p = < 0.001, respectively) Hierarchical linear regressions identified R2 = 0.2 (p = < 0.001) for self-stigma content independently predicting depression, and also R2 = 0.38 (p = < 0.001) for self-stigma content and self-stigma process both independently predicting depression
	Mak and Kwok (2010); Hong Kong, China	To investigate the relationships between social support, affiliate stigma and psychological well-being among parents of children with autism	Quantitative cross-sectional questionnaire survey	Purposive sampling	188 parents (mean age = 42.6 years), 84.3% of whom were female, 89.1% were married, and 72.8% had a secondary school educational level. 37.8% of their children also had an intellectual disability. 88.3% were male children	Courtesy stigma, affiliate stigma	Courtesy stigma—7 items from ‘Devaluation of Consumer Families Scale’ (Struening et al. 2001) plus following 2 items: “most people would not be friends with parents who have children with ASD” and “most people stigmatise parents of children with ASD” Affiliate stigma—‘Affiliate Stigma Scale’ (Mak and Cheung 2008)	Psychological wellbeing measured using the ‘Psychological Well-being’ subscale of the ‘Mental Health Inventory’ (Veit and Ware 1983)	Courtesy stigma and affiliate stigma significantly negatively correlated with psychological wellbeing (r = − 0.26, p = < 0.01, r = − .47, p = < 0.01)
	Wong et al. (2016); Hong Kong, China	To examine the associations between affiliate stigma, self-compassion, and psychological distress among Chinese parents of children with autism	Quantitative cross-sectional questionnaire survey	Purposive sampling	180 out of 600 parents participated (RR = 39%). Participants’ mean age was 42.3 years, 84.2% of whom were female, 87.2% married and 49.2% educated to senior secondary school level. Their children (n = 180; mean age = 9.8 years) were mostly male (84.2%) and mostly attended a mainstream school (54.7%)	Affiliate stigma	‘Affiliate Stigma Scale’ (Mak and Cheung 2008)	Psychological distress measured using the ‘Psychological Well-being’ subscale of the ‘Mental Health Inventory’ (Veit and Ware 1983)	Affiliate stigma significantly positively correlated with psychological distress (r = 0.44, p = < 0.01). Significant association between affiliate stigma and psychological distress among caregivers with low levels of self compassion (β = 0.26, t = 3.33, p < .01) but not among their counterparts with high levels of self-compassion (β = 0.02, t = 0.28, p = .78)
Middle-Eastern	Werner and Shulman (2013); Israel	To examine the relationship between affiliate stigma and caregivers’ subjective well-being among family caregivers of individuals with intellectual disability, autism or physical disabilities	Quantitative cross-sectional questionnaire survey	Purposive sampling	170 caregivers (mean age = 43.2 years), 78.8% of whom were female and 57.6% of whom described themselves as ‘secular’. Their children (n = 170; mean age = 1.9 years) were mostly male (61.8%) and mostly attended a special education school (63.5%). 32.9% had a diagnosis of autism, 22.4% intellectual disability and 27.1% a physical disability	Affiliate stigma	‘Affiliate Stigma Scale’ (Mak and Cheung, 2008)	Subjective well-being measured using the ‘Personal Well-being Index’ (International Wellbeing Group, 2006)	Affiliate stigma significantly negatively correlated with autism caregivers’ subjective wellbeing compared with other diagnoses (B = − 0.20, p < .05). Positive meaning in caregiving, self-esteem, social support and low burden buffered/protected against affiliate stigma’s relationship with negative subjective wellbeing among autism caregivers
	Dababnah and Parish, (2013); Palestinian Arabs living in the West Bank villages and refugee camps	To examine parents of children with autism’s knowledge, attitudes, burdens and coping strategies	Qualitative focus groups and one-to-one interviews	Purposive sampling	24 parents (20 mothers) of 24 children with autism aged between 4 and 17 years (mean age = 10 years), 16 of whom were boys. According to parents’ own characterisations, 7 children had ‘severe behavioural problems’, and 4 were ‘high functioning’	Public stigma, courtesy stigma (e.g. parent blaming), stigma from health professionals (e.g. being denied services), affiliate stigma (e.g. embarrassment and personal shame about their child’s condition)	Study authors’ interpretations	Study authors’ interpretations	Respondents mentioned frustration, anger and depression resulting from a child being denied services due to their autistic symptoms. These consistent frustrations weighed on parents. One mother described having chronic headache from her daughter’s condition. Some participants reported that they presented a stoic face to outsiders, but continued to silently suffer In total, these daunting financial psychological, and caregiving burdens led parents to feel confined to their homes, embarrassed about their child’s condition, and desperate for answers
	Crabtree (2007); Sharjah, United Arab Emirates	To explore the lived experience of care-giving families of children with developmental disabilities receiving specialised day services	Ethnographic approach utilising longitudinal qualitative semi-structured interviews	Purposive sampling	15 parents (mainly mothers) from diverse ethnic and socio-economic backgrounds. Four women were divorced or widowed. Age range of disabled children ranged from 4 to 16 years, just over half were boys. Six children were formally diagnosed with autism	Public stigma, courtesy stigma (‘mother blaming’) and stigma from health professionals	Study author’s interpretations	Study author’s interpretations	The experiences of stigma left Emirati women feeling vulnerable, including feelings of blacklisting by medical authorities that were “a source of tremendous stress and anxiety”. Some cases where parents’ piety led to the view that their child’s birth and disability were curses or punishments from Allah, leading to “feelings of hopelessness and depression and in some cases accompanied by covert or overt rejection of the child.”
	Dehvani et al. (2011); Isfahan and Shahr-e Kord cities, Iran	To investigate the relationship between internalised stigma in mothers and ‘autism quotient’ in relation to the mental health of mothers of children with autism	Quantitative cross-sectional questionnaire survey	Purposive sampling	95 mothers (mean age = 29.5 years) of autistic children (mean age = 7.7) enrolled at a centre of special education, 79% of whom were male	Affiliate stigma	‘Affiliate Stigma Scale’ (Mak and Cheung 2008)	Depression, anxiety, somatic symptoms, and general mental health all measured using the General Health Questionnaire-28 (Goldberg, 1972)	Affiliate stigma significantly predicted general mental health (r = 0.54 [sig = 0.00], adjusted R2 = 0.27)
Western	Resch et al. (2010); Seven communities across a southwestern state in the United States	To identify specific sources of challenges related to raising a child with a disability as expressed by parents	Qualitative focus groups	Purposive sampling	40 parents (36 of whom were mothers) of mainly Anglo (n = 22) and Hispanic (n = 11) ethnicity living in urban (n = 15), rural (n = 13) and suburban (n = 11) communities. Eight of this cohort’s described their child’s ‘primary disability’ to be autism, and the majority stated their children’s primary classroom setting to be ‘regular’ (n = 17) or ‘self-contained’ (n = 18)	Public stigma, school and community social exclusion	Study authors’ interpretations confirmed by parent representatives in a respondent validation exercise. The interview guide included the directly relevant questions about stressors and challenges	Study authors’ interpretations confirmed by parent representatives in a respondent validation exercise. The interview guide included the directly relevant questions about stressors and challenges	The experience of school and community exclusion including the adverse reactions of some in the community to having their children included in community activities was one of four major themes that the authors identified as influencing parent wellbeing (and supported during the respondent validation exercise), a concept the authors define in the study as including mental health
	Broady et al. (2018); Sydney and the South Coast regions of New South Wales, Australia	To investigate the lived experience of stigma among carers of children with high functioning autism	Qualitative exploratory semi-structured one-to-one interviews	Convenience sampling	15 carers (9 female, 6 male) of children diagnosed with HFA. 12 participants were married, 1 participant was separated and 2 were divorced. Their children were aged from 5 to 19 years (mean age = 10 years). The time since receiving a diagnosis of HFA ranged from 6 months to 11 years (mean = 4.86 years, SD = 3.79 years)	Public stigma, courtesy stigma, professional (school teachers)	Study authors’ interpretations	Study authors’ interpretations	Felt and enacted stigma was experienced through rejection and judgement from family and friends which was particularly hurtful. Rejection from wider public was often met with a sense of anger, while rejection from family and friends led to a much more significant and emotionally charged impact, leading carers’ to alter behaviour through strategies such as social withdrawal. Authors conclude that perceptions of stigma are associated with poor subjective wellbeing
	Gray (2002); Brisbane and its adjacent coastal regions, Australia	To examine the social experiences of families of children with autism	Ethnographic approach utilising retrospective longitudinal qualitative semi-structured interviews	Purposive sampling from a cohort who had previously participated in a quantitative survey and qualitative study 10 years prior	28 parents (19 mothers and 9 fathers representing 20 families) of 20 individuals with autism (13 males), aged 13 to 27 years. 8 were attending a special school, 8 were receiving community service support, 2 were in residential care and 2 were not receiving any services	Public stigma, courtesy stigma (e.g. grandparents blaming parents)	Study author’s interpretations	Study author’s interpretations. The interview schedule included a directly relevant question about “the effects of the situation on the parent’s health”	Parents were still being affected by depression, anxiety and anger associated with “substantial amounts of self-reported psychological distress” among parents but particularly among mothers. The study author interprets this as partly due to an “increased exposure to negative social reactions by outsiders.” However, compared to their experiences 10 years previously, many parents had become less “sensitive to the reactions of outsiders and find stigmatizing behaviors less threatening to their self-esteem. Although several parents claimed that social rejection still disturbed them when it occurred, it did not seem to have the emotional impact on them that it did previously”

### Quality Appraisal

All of the qualitative studies demonstrated clear congruity between their respective methodologies and their research question(s)/objective(s), data collection methods, analysis and representation of data (including participant voices being adequately represented), and interpretation of results (which aligned well with their respective conclusions). However, only two studies showed clear congruity between their philosophical or theoretical perspectives and research methodology, with both adopting an ethnographic theoretical perspective and adopting methods in keeping with this approach (Crabtree 2007; Gray 2002). Three others provided clear evidence of ethics approval by an appropriate body (Resch et al. 2010; Dababnah and Parish 2013; Broady et al. 2018). Only Crabtree (2007) provided a statement locating the researcher culturally or theoretically in the study, and addressed the influence of the researcher on the study. Overall, the five qualitative studies all scored well and, in the case of Crabtree (2007), very well.

Regarding the quantitative studies, all seven employed valid measures for the identification of both stigma and mental health, with these outcomes also being measured in a standard, reliable way for all participants. Three studies utilised an appropriate sampling frame for their respective target populations and sampled their participants in an appropriate way by attempting to recruit everybody eligible within their supporting clusters (Mak and Kwok 2010; Werner and Shulman 2013; Wong et al. 2016), none employed true random sampling (it was not made clear how clusters were recruited and if they adequately represented the wider target population) and therefore none constructed an adequate sample size. Because of this and that no studies provided sufficient clarity on the characteristics of the target population, ruling out sample coverage bias for these studies was not possible. Mak and Kwok (2010) and Wong et al. (2016) reported a response rate but it was unclear if either were adequate for their respective target populations. Overall, the studies conducted by Mak and Kwok (2010), Werner and Shulman (2013) and Wong et al. (2016) were the most methodologically robust. A full breakdown of the quality appraisal results is presented in Table 2.

**Table 2:** Quality appraisal of included studies

	Congruity between philosophy and methodology?	Congruity between methodology and research question or objectives?	Congruity between methodology and data collection methods?	Congruity between methodology and representation and data analysis?	Congruity between methodology and interpretation of results?	Is there a statement locating the researcher culturally or theoretically?	Is the researchers’ influence on the research, and vice- versa, addressed?	Are participants, and their voices, adequately represented?	Is there evidence of ethical approval by an appropriate body?	Do the conclusions flow from the analysis, or interpretation, of the data?	Overall quality score
Qualitative											
Crabtree (2007)	1	1	1	1	1	1	1	1	0	1	9
Gray (2002)	1	1	1	1	1	0	0	1	0	1	7
Resch et al. (2010)	0	1	1	1	1	0	0	1	1	1	7
Dababnah and Parish (2013)	0	1	1	1	1	0	0	1	1	1	7
Broady et al. (2018)	0	1	1	1	1	0	0	1	1	1	7

	Was the sample frame appropriate to address the target population?	Were study participants sampled in an appropriate way?	Was the sample size adequate?	Were the study subjects and the setting described in detail?	Was the data analysis conducted with sufficient coverage of the identified sample?	Were valid methods used for the identification of the condition?	Was the condition measured in a standard, reliable way for all participants?	Was there appropriate statistical analysis?	Was the response rate adequate, and if not, was the low response rate managed appropriately?	Overall quality score	
Quantitative											
Chan and Lam (2017)	0	0	0	1	0	1	1	1	0	4	
Dehvani et al. (2011)	0	0	0	0	0	1	1	0	0	2	
Mak and Kwok (2010)	1	1	0	1	0	1	1	1	0	6	
Werner and Shulman (2013)	1	1	0	1	0	1	1	1	0	6	
Wong et al. (2016)	1	1	0	1	0	1	1	1	0	6	
Ting et al. (2018)	0	0	0	1	0	1	1	1	0	4	
Chan and Lam (2018)	0	0	0	1	0	1	1	1	0	4	

Key: 1 =  Yes; 0 =  No/unclear

### Stigma and Informal Caregiver Mental Health—East Asian Cultures

Ting et al. (2018) quantitatively investigated the relationship between affiliate stigma and depression among 263 caregivers of autistic children in Mainland China. Affiliate stigma was found to be significantly positively correlated with depression (r = 0.55, p = < 0.01). After integrating the results from path analyses on two models, caregivers low in self-esteem, high in proneness to shame, with poor family functioning (particularly family adaptability) were found to be significantly more likely to experience affiliate stigma and have more depressive symptoms.

In one of four studies located in Hong Kong, Chan and Lam (2017) found, among a large sample of parents (n = 424), public stigma to be significantly correlated with depression (r = 0.27, p = < 0.001) and anxiety (r = 0.23, p = < 0.001) in positive directions, as was courtesy stigma (r = 0.32, p = < 0.001; r = 0.47, p = < 0.001 respectively). ‘Trait mindfulness’ (the tendency to be mindful in daily life) was found to significantly moderate the relationship between both forms of stigma with depression and anxiety. The authors interpreted this to be because mindful parents were better able to regulate their emotions in the face of stressors such as stigma which helped to reduce the intensity and duration of the subsequent emotional impact.

Using the same sample as above, Chan and Lam (2018) also tested the psychometric properties of a recently developed measure of self-stigma process, the Self-Stigmatizing Thinking’s Automaticity and Repetition Scale (STARS). As part of this exercise, they assessed both the concepts of ‘self-stigma content’ (the extent to which caregivers endorse their self-stigmatizing thoughts) and ‘self-stigma process’ (the extent to which they think about their self-stigmatizing thoughts repetitively and automatically as a mental habit) in relation to various outcomes including caregiver depression. They found that both concepts significantly positively correlated with depression (content: r = 0.45, p = < 0.001; process: r = 0.61, p = < 0.001). Their hierarchical linear regressions of these self-stigma concepts and their independent predictive power of caregiver depression identified R2 = 0.2 (p = < 0.001) and R2 = 0.38 (p = < 0.001) model strength for self-stigma content (block 1) and self-stigma content and process (block 2) respectively.

Mak and Kwok’s (2010) Hong Kong-based study of 188 parents of autistic children assessed the relationship between psychological wellbeing and stigma, finding a significant negative correlation between both psychological wellbeing and courtesy stigma (r = − 0.26, p = < 0.01) and affiliate stigma (r = − 0.47, p = < 0.01). They also tested indirect pathways between stigma and psychological well-bring wellbeing via perceived controllability, self-blame, and social and professional support, finding that these variables explained 40.2% of the variance.

Wong et al. (2016)’s Hong-Kong based study also assessed affiliate stigma’s relationship with psychological distress among parents of autistic children (n = 180), identifying a significant positive correlation (r = 0.44, p = < 0.01). They also examined the role of self-compassion, finding a significant association between affiliate stigma and psychological distress among parents with low levels of self-compassion but not among those with high levels of self-compassion. The authors state that this is because self-compassionate parents are less likely to be self-critical, less likely to hold negative thoughts and feelings, and more likely to experience self-acceptance and hopefulness for the future, all of which helps to combat the feelings of shame that affiliate stigma produces.

### Stigma and Informal Caregiver Mental Health—Middle-Eastern Cultures

Werner and Shulman (2013)’s assessment of affiliate stigma’s relationship with caregiver wellbeing among 170 informal caregivers of children with autism (n = 56), intellectual disability (n = 38) or physical disabilities (n = 76) residing in Israel also identified a statistically significant negative association between these concepts both across the total sample (r = − 0.58, p < .001) and when diagnosis was controlled for in their regression analyses (autism vs other diagnoses: B= − 0.20, p = < .05). The authors state that among caregivers of autistic people, affiliate stigma was positively associated with lower ratings of wellbeing, whereas no such association was identified among caregivers of individuals with other diagnoses. Their regression analyses also found that, among autism caregivers compared with other diagnoses, the significant association between affiliate stigma and caregiver wellbeing disappeared when positive meaning in caregiving, self-esteem, social support and caregiver burden were controlled for, leading the authors to conclude that these psychosocial variables act as a buffer against the negative influence of stigma on caregiver well-being. The authors also conclude that their results stress the importance of helping families to form and maintain social support, as well as a positive perspective from which they use to make find positive meaning in their lives.

A qualitative study conducted by Dababnah and Parish (2013) investigated parents of autistic children’s knowledge, attitudes, burdens and coping strategies. The study, which sampled 24 Palestinian Arab parents (mainly mothers) living in West Bank villages and refugee camps, reported that “discrimination and stigma from extended family members and the larger community intensified parents’ feelings of shame and experiences of social isolation” (p. 1670) and that parents experienced frustration, anger and depression. This was for many reasons including their children being denied services which consistently occurred and weighed heavily on parents. Some participants reported that they “presented a stoic face to outsiders, but continued to silently suffer” (p. 1675). This linked to perceptions of having a child with a disability as a “sign of shame”, with many parents admitting to actively hiding their situation from others in their community. This was particularly distressing for parents due to the social nature of Palestinian life, with one mother, crying, stating, “I become withdrawn because I do not want to feel embarrassed in front of people. [My son] is very active and when people come over…. it is not very nice” (p. 1673). One parent, blaming public stigma, relayed: “You can see that we [parents] can talk about these things, because we are not ashamed that we have kids with special needs, but look at our society. I cannot say that I have a girl with special needs. This is my problem that I have to hide this fact” (p. 1673). Parents also reported incidents of courtesy stigma, such as people on the bus chastising a mother for her “inability to teach her child to ‘differentiate between right and wrong’” (p. 1674). A third of parents also described substantial problems in finding acceptance from their families, with one mother stating, “Some people from my family were understanding. Others were not. They were telling us to get rid of him. To get rid of him! [They said], ‘Why are you taking care of him?’ That is how some people think. When he got sick, they kept telling us, ‘Why are you even spending money on him?’” (p. 1674). The authors report that, in sum, “the daunting financial, psychological, and caregiving burdens led parents to feel confined to their homes, embarrassed about their child’s condition” and that “while the parents demonstrated remarkable resilience, the findings overall revealed them to be incredibly vulnerable to further psychological, emotional and financial stressors” (p. 1676).

Crabtree (2007) collected qualitative data over a 10 month period after interviewing informal caregivers about their lived experience of caring for a child with developmental disabilities several times (this included caregivers of six children with a diagnosis of autism). The study participants, whose children were receiving specialised day services in Sharjah, United Arab Emirates, reported that the public stigma associated with the birth of disabled children leaves Emirati women feeling vulnerable and rejected. For example, one participant felt resigned to remaining single due to the stigma associated with having a young autistic child. Some parents, including mothers of autistic children (as well as those caring for children with other disabilities), described feeling blacklisted by medical authorities due to being negatively labelled and stigmatised as someone unable to give birth to healthy children, with such stigma being “a source of tremendous stress and anxiety” (p. 54). The study also identified cases, including among autism caregivers specifically, where parents’ piety led to the view that their child’s birth were curses or punishments from Allah, leading to “feelings of hopelessness and depression and in some cases accompanied by covert or overt rejection of the child” (p. 55). However, this type of divine punishment attribution was generally ascribed to the fathers by the mothers, leading to a lack of family acceptance towards their autistic child.

Dehvani et al. (2011) assessed affiliate stigma’s relationship with mental health as part of their investigation of internalised stigma among mothers (n = 95) living in the Iranian cities of Isfahan and Shahr-e Kord and whose autistic children attended a centre of special education. Their analysis also demonstrated a significant positive association between internalised stigma and poor mental health (r = 0.54, sig = 0.00, adjusted R2 = 0.27). They also examined the potential moderating effect of ‘autism quotient’ [as measured by the Gilliam Autism Rating Scale (Gilliam 2001)] but found no significant association.

### Stigma and Informal Caregiver Mental Health—Western Cultures

Resch et al. (2010) qualitatively investigated the experiences of forty parents from urban, rural, and suburban communities across the South-West of the United States. This included eight parents of autistic children (the largest sub-group of disability sampled) who identified parental experiences of exclusion within the school setting and the wider public, with one father, describing his autistic son’s struggle to be included in a baseball league stating, “You do have some people that have strong opinions like, ‘they’re not supposed to be here, they’re not supposed to be included with us as a group’” (p. 144). The experience of such stigma was one of four major themes that the authors identified as key to influencing parents’ mental health.

Broady et al. (2018) conducted one-to-one semi structured qualitative interviews with 15 family caregivers (9 mothers, 6 fathers) of high functioning autistic children living in Sydney and the South Coast regions of New South Wales, Australia, in order to explore caregivers’ lived experience of stigma. Four domains of stigmatising experience were identified-lack of knowledge, judgement, rejection and lack of support—with each domain existing in unique and nuanced ways across public and school contexts, family and friends. The felt and enacted stigma caregivers experienced through rejection and judgement from family and friends were particularly hurtful. Rejection from wider public was often met with a sense of anger, while rejection from family and friends led to a much more significant and emotionally charged impact, leading caregivers’ to alter their behaviour through strategies such as social withdrawal. The study authors conclude that perceptions of stigma are associated with poor subjective wellbeing.

Gray’s (2002) 10-year follow-up qualitative study of 28 families of autistic children’s social experiences living in and around Brisbane, Australia, identified substantial amounts of self-reported psychological distress among parents, particularly mothers, and that “slightly over half of the parents reported significant degrees of anxiety and depression and approximately a third of these were receiving psychotherapy and/or medication to cope” (p. 218). Gray interpreted this as partly due to public stigma [an “increased exposure to negative social reactions by outsiders” (p. 218)]. While poor mental health was therefore prevalent, compared to their experiences 10 years previously, many parents had become less “sensitive to the reactions of outsiders and find stigmatizing behaviors less threatening to their self-esteem. Although social rejection still disturbed them when it occurred, it did not seem to have the emotional impact on them that it did previously” (p. 221). Gray also reported that courtesy stigma from extended families, especially grandparents, persisted for some although negative perceptions, such as being critical of parents’ child raising skills and frequent denials of their grandchild having a disability, had largely subsided, in part due to the parents’ developing confidence in both their child’s diagnosis and their own parenting abilities.

## Discussion

All twelve studies produced evidence pertaining to the harmful relationship between stigma and mental health among informal caregivers. This included stigma being related to depression (Crabtree 2007; Gray 2002; Dababnah and Parish 2013; Chan and Lam 2017, 2018; Ting et al. 2018), anxiety (Gray 2002; Chan and Lam 2017), psychological distress (Gray 2002; Wong et al. 2016), psychological wellbeing (Broady et al. 2018; Mak and Kwok 2010; Werner and Shulman 2013), psychological burden (Dababnah and Parish 2013) and general mental health (Dehvani et al. 2011).

While the rigour of these studies varied (particularly among quantitative studies), given the consistency of this finding, which remained clear across diverse study designs and a wide range of socio-demographic and cultural settings [i.e. shared ways of living among a group of people that includes beliefs, values, ideas, language, communication, and norms (Papadopoulos 2006)], it can be concluded with confidence that an association between autism stigma and informal caregiver mental health exists. This held true for each form of stigma identified which included public stigma, courtesy stigma, affiliate stigma (including self-stigma content and process) and stigma from professionals. For the latter, this was identified among health (Crabtree 2007; Resch et al. 2010; Dababnah and Parish 2013) and school authorities (Resch et al. 2010; Broady et al. 2018).

**Fig. 2:**
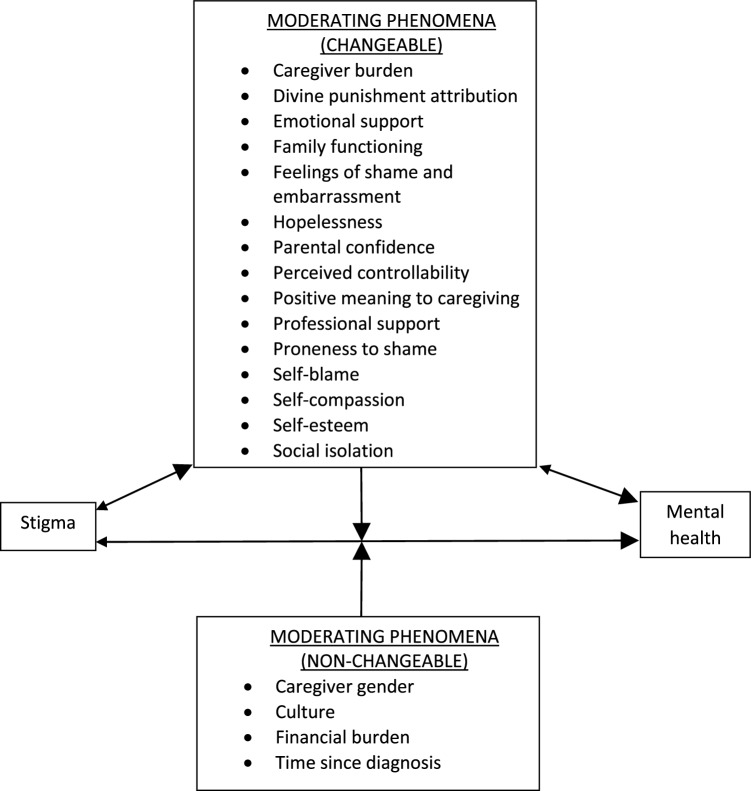
Theoretical framework of the relationship between autism stigma and informal caregiver mental health

There also appears to be a complex relationship between stigma and mental health which is not necessarily always one-directional (stigma leading to poor mental health) or even bi-directional (stigma leading to poor mental health and vice-versa). Indeed, the review identified a range of phenomena that moderated the strength of the relationship between stigma and mental health outcomes among caregivers of autistic individuals. There are represented in Fig. 2 where a theoretical framework that synthesises all the identified evidence is proposed. The inter-relationships that exist between these variables are likely to be ever-unfolding and complex. For example, it is likely that informal caregivers who experience both stigma and financial burden are particularly vulnerable to social isolation (as reported by Dababhna and Parish 2013) which may hinder access to support, intensifying the overall negative affect upon mental health. Further, since many of the identified moderating phenomena relate to the construct of depression (e.g. feelings of shame, hopelessness, self-blame, self-esteem), it is reasonable to expect that informal caregivers who have a vulnerability and/or a history of experiencing mental health problems may be particularly prone to experiencing stigma and its negative effects. It remains unknown if and how the strength of reported associations between stigma and mental health changes after controlling for variables such as history of depression or other mental health problems. Future quantitative research could control for these and other potential confounding variables, for example, through statistical regression modelling (McNamee 2005).

In theory, anti-stigma interventions are most likely to result in greater impact if they target at least one of the changeable moderating phenomena (i.e. phenomena that can be changed through research intervention) among caregivers that intersect with at least one of the non-changeable moderating phenomena (i.e. phenomena that cannot be changed through research intervention). For example, interventions that target boosting self-esteem and self-compassion (changeable phenomena) among caregivers of recently diagnosed children living in socio-cultural settings that are particularly vulnerable to stigmatising attitudes (non-changeable phenomena) are likely to have impact. Indeed, tailoring interventions according to how local culture influences the production of stigmatising attitudes is likely to be particularly effective. There are several reasons for this. First, knowledge and understanding about autism varies cross-culturally, meaning that different cultural groups are likely to hold different types of misconceptions about autism. This has been demonstrated by Obeid et al. (2015) who showed that while University student groups in the United States (n = 346) and Lebanon (n = 329) both held misconceptions about autism, the types of misconceptions they held were significantly different. For example, the Lebanese students were much more likely to believe that autistic people are deliberately uncooperative, and that autism is caused by negative parenting, whereas the US students more frequently believed that all autistic people had learning difficulties. Second, there is evidence that the way in which stigma manifests in collectivist, group-based cultures (which place priority on community interdependence and shared group norms and values) differs to individualist cultures (which place priority on personal independence, goals and values) (Papadopoulos et al. 2013; Papadopoulos 2016b). For example, Ting et al (2018) and Chiu et al (2013) argue that in the Chinese culture, parents of autistic children are particularly vulnerable to experiencing affiliate stigma because of the sense of shame people have had ingrained in them as a result of their socialisation within a group-focused, collectivist culture. This can also be identified in Dababhna and Parish’s (2013) study of collectivist Palestinian culture in which stigma from the extended family members and wider community increased parents’ feelings of shame and likelihood social isolation. This type of stigma that is rooted in the fear of shame from an in-group was not observed in the studies set in the Western cultural contexts. On the contrary, Broady et al. (2018) study of family caregivers in Australia reported that negative judgements from people inside their collective of family and friends associated with feelings of hurt rather than shame and embarrassment. Religion also plays a key role in how and why autism stigma is rooted in culture. For example, Crabtree’s (2007) study reported cases where parents’ religious piety led to the view that their child’s birth were punishments from Allah. Alqahtani (2012) and Bankole (2016) have previously highlighted how religion plays a prominent role in shaping people’s cultural values and perceptions about autism, particularly when professional autism services do not exist, are inaccessible and/or distrusted. This can, for example, lead to people attributing autism to the ‘evil eye’ (ascribing one’s misfortunes to ‘envy in the eye of the beholder’) and as such turning to religious intervention and exorcism. Similarly, in communities where high quality professional services and resources are less available, we should also expect to observe poorer knowledge about autism and thus higher rates of autism stigma (Grinker et al. 2011).

Focusing upon new caregivers is also important since they are likely to be particularly vulnerable to self-blame during the early stages of diagnosis and, as such, to affiliate stigma. As this may be the first time they have encountered autism, they may be prone to misconceptions, myths, and negative stereotypes, and they may not yet realise how valuable and rewarding the caregiving role can be. Instead, new caregivers may be experiencing shock from a diagnosis they were unprepared for, leaving them psychologically vulnerable, and without the new social support networks that require time to develop. This was highlighted by Gray (2002) who stated that a key reason why the impact of stigma upon caregiver mental health generally declined over time in his study was because over time parents had formed new, trusted friends who accepted their child’s disability.

Boosting informal caregiver mental health is also likely to have a positive reverse effect on the changeable moderating phenomena (e.g. reducing feelings of hopelessness, increased parental confidence, proneness to shame), as well as boosting resilience towards stigma. However, given the findings in this review, the reverse direction of this relationship is likely to be more powerful (as represented in Fig. 2 by the arrow sizes). This can be seen within the qualitative studies where accounts of stigma and the moderating phenomena were described as contributors to poor mental health. It is also important to note the proposed framework should not be viewed as an exhaustive representation of moderators given that this remains an under-researched area. Further, while targeting the moderating variables described in our theoretical framework may yield most impact, the challenge associated with achieving this is likely to be particularly difficult given how complex and sophisticated interventions will need to be to moderate such variables.

While many stigma-protecting interventions have been produced and tested among other populations (e.g. people with severe mental health problems), none have yet been produced for the autism caregiver population. There have been, however, several recent initiatives aimed at reducing the stigma towards autism more generally. For example, a ‘Sesame Street’ initiative titled, ‘See Amazing in All Children’ aims to combat the stigma (and isolation) often experienced by autistic children and their families (Sesame Workshop, No date). Georgetown University’s (2017) evaluation of the website’s impact upon knowledge, acceptance and positive attitudes showed small to moderate positive gains in all areas across 1 week [for parents of non-autistic children (n = 698)] and 1 month [for parents of autistic children (n = 331)] periods. The evaluation measured ‘feelings of strain to caregiving’ (assessing issues such as worries about the future and feeling tired) which was negatively associated with parents of autistic children’s beliefs about their parental competence. In the evaluation report, the authors state that their findings underline that autistic children are more likely to thrive when their family members possess better mental health. This substantiates the importance of the caregiver mental health and partially supports the inclusion of ‘parental confidence’ as a moderating variable in our theoretical framework.

There are several limitations in this review. First, it is possible that not all eligible studies were identified, particularly since not all specific mental health outcomes of interest (e.g. ‘depression’) were added to the keyword search strategy. Second, the included studies all had some level of internal bias. For example, none of the quantitative studies produced generalisable samples or reported adequate response rates. Future quantitative studies should therefore focus upon this through conducting sample size calculations and maximising (and clearly reporting) response rates. The main methodological weakness identified across the qualitative studies was the lack of any statement locating the researchers culturally or theoretically. It would be useful to know, for example, if investigators were ‘insider researchers’ and the impact this may have had. In the current study, the lead researcher is a father of an autistic child; this may help with interpreting the importance of the study findings and their implications. It may also potentially undermine objectivity but critically examining this potential issue at the outset helped negate this concern. Overall, while the synthesised pool of evidence demonstrated reasonable robustness, stronger methodological studies are required, as are studies with quantitative longitudinal designs and, in particular, experimental studies. This would help ascertain how the nature of the statistical relationship between stigma and mental health changes over time, and pave the way for an understanding of which moderating variables play key predictive roles across different settings. There are other key limitations in the evidence pool. First, parents were the predominant focus of the included studies. Developing an understanding of how stigma affects other types of informal caregivers, such as grandparents and siblings, is important. It also remains unknown if and how the theoretical framework might need tailoring for autistic parents (none of the included studies assessed to what extent their caregiver samples consisted of autistic individuals). Furthermore, systematically investigating the influence that comorbidities, such as fragile X syndrome or epilepsy, has upon the relationship between experiencing stigma and mental health among caregivers of autistic people would be helpful in refining our theoretical framework. Second, our framework collapses all forms of identified stigma into one higher-level autism stigma concept. This was conducted due to the current limited pool of research evidence available. However, as these concepts are not homogenous, and as the evidence pool in this area increases, the framework’s precision can be enhanced by specifying each stigma concept individually. This will augment the precision of interventions. The same point can be made in relation to ‘mental health’; as research increases, the disaggregation of the mental health concept should boost precision. Third, with the exception of Gray (2002), all studies focused upon parents of young children (aged under 18). Fourth, there are many socio-cultural settings that have not yet been studied (e.g. no studies took place across the African or South American continents). Increasing the amount, quality and diversity of research studies in this area will produce the comprehensive understanding that practitioners and policy makers require to base successful, impactful approaches upon.

## Conclusion

This review’s findings confirm there to be an association between autism-related stigma and informal caregiver mental health. Given the importance of caregiver mental health in supporting those who they are caring for, and until public autism stigma is eradicated, interventions which help protect caregivers from autism stigma are necessary. Therefore, future caregiver-specific interventions should strategically target the moderating variables described in this study’s theoretical framework at the caregiver level to support and protect this population from different forms of autism stigma so that their mental health can be strengthened. Interventions that are able to produce a long-term positive effect upon mental health, for example, by constructing long-lasting support structures and providing informal caregivers with practical skills they can take forwards (for example, self-compassion and mindfulness techniques, and having ready-made responses for when they encounter stigma), will be particularly powerful. However, given that stigma-focused interventions have not yet been produced, the focus of future interventions should be to first assess their feasibility and acceptability. Interventions will also be enhanced if they are designed, developed and implemented in a participatory approach with caregivers and autistic individuals in a fashion that is genuine rather than tokenistic (Pellicano et al. 2014).

This review also has implications for professional practice. First, it highlights the importance of professionals who work with autistic individuals and their families to be mindful of the impact autism stigma may have on mental health, and the presence of any moderating phenomena described in the theoretical framework. This can enable professionals to be better equipped in minimising stigma’s impact and intervening early. For example, professionals who can quickly identify low parental confidence, feelings of hopelessness and social isolation can intervene early by providing and/or recommending sources of support. Further, the review highlights the negative impact of perceived stigma from professionals on caregivers. Therefore, professionals should critically reflect and discuss with their peers their own attitudes, perceptions and practices to ensure they are serving their clients in a non-stigmatising fashion. Holding negative attitudes jeopardises quality of care (Zheng et al. 2014) and may deter families from accessing services. Finally, given how autism stigma manifests in different degrees and types across cultures, it will be of benefit to families if the professionals they access are culturally competent; otherwise they may be rejected and/or distrusted.

Finally, further, rigorous research across different socio-cultural settings and groups not previously or recently investigated (since cultures are dynamic and ever-changing) are required, especially given how crucial tailoring interventions to particular cultural settings is likely to be. This would boost the size and quality of the evidence pool in this area, critical in building and further refining this review’s proposed theoretical framework which represents a start to comprehensively understanding the interconnectedness of the phenomena identified. It would also mean enabling us to capture a wider range of perspectives and voices, many of which are currently not being captured in the literature that examines the relationship between autism stigma and caregiver mental health. Through such means, producing effective interventions, policies and practice can become achievable so that the wellbeing of informal caregivers and those who they are caring for can be supported.
